# O6-3 Analysis of the local health-enhancing physical activity policies in the French Riviera

**DOI:** 10.1093/eurpub/ckac094.043

**Published:** 2022-08-29

**Authors:** Antoine Noël-Racine, Jean-Marie Garbarino, Bernard Massiera, Anne Vuillemin

**Affiliations:** Université Côte d'Azur, LAMHESS, France

**Keywords:** health-enhancing physical activity, local policy, municipality

## Abstract

**Background:**

According to the socio-ecological model, policy is one of the levers to initiate structural and environmental changes to foster health-enhancing physical activity (HEPA) promotion (Golden et al., 2015). However, little is known about local evidence to support governments in their policies to promote HEPA (Bull, 2018). Research on HEPA policies at local level seems particularly remains scant (Noël Racine et al., 2020). This study aims to carry out the collection of comprehensive information on municipal HEPA policies of the French Riviera to provide an overview of the development of these policies on this territory.

**Methods:**

Mid-size cities from 2 counties of the French South Region were targeted (n = 17). In each city, a local HEPA policy analysis tool, CAPLA-Santé, was administered to key informants heading the departments of sport, health and social. CAPLA-Santé is a local policy analysis tool adapted from the national HEPA policy analysis tool (HEPA PAT version 2) developed by the World Health Organization. Data were collected through semi-structured interviews and documents analysis. These empirical data were used to make an inductive analysis.

**Results:**

A total of 10 mid-size cities were volunteered to participate. Key informants from sport (n = 10), health (n = 5) and social (n = 6) departments were interviewed. Several written HEPA policies were formalized in 6 cities, 4 of them based their policies on scientific evidences or a national policy. These policies (n = 14), were mainly from the sport (n = 8) and the health sectors (n = 4). Some cities had a department head to ensure cross-sectoral collaboration (n = 3). Key informants reported that the support of national policies, the commitment of elected officials and an important local stakeholders' network could facilitate the HEPA promotion; whereas lack of intersectoral collaboration and resources could be a limitation.

**Conclusions:**

The results help to better understand the local HEPA policies, highlighting some barriers, facilitators and perspectives. Using a local HEPA policy analysis tool can provide evidence to support policymakers. These findings could be valuable to scale up the HEPA promotion at the local level.

